# Supplementary material for: Evaluation of the Impact of Near-Infrared Multiwavelength Locked System Laser Therapy on Skin Microbiome in Atopic Dogs
Source: Animals (Basel). 2024 Mar 14;14(6):906. doi: 10.3390/ani14060906 (PMC10967621; doi:10.3390/ani14060906)
Supplement: Supplementary file 1 [file animals-14-00906-s001.zip › animals-2880330-supplementary.pdf]

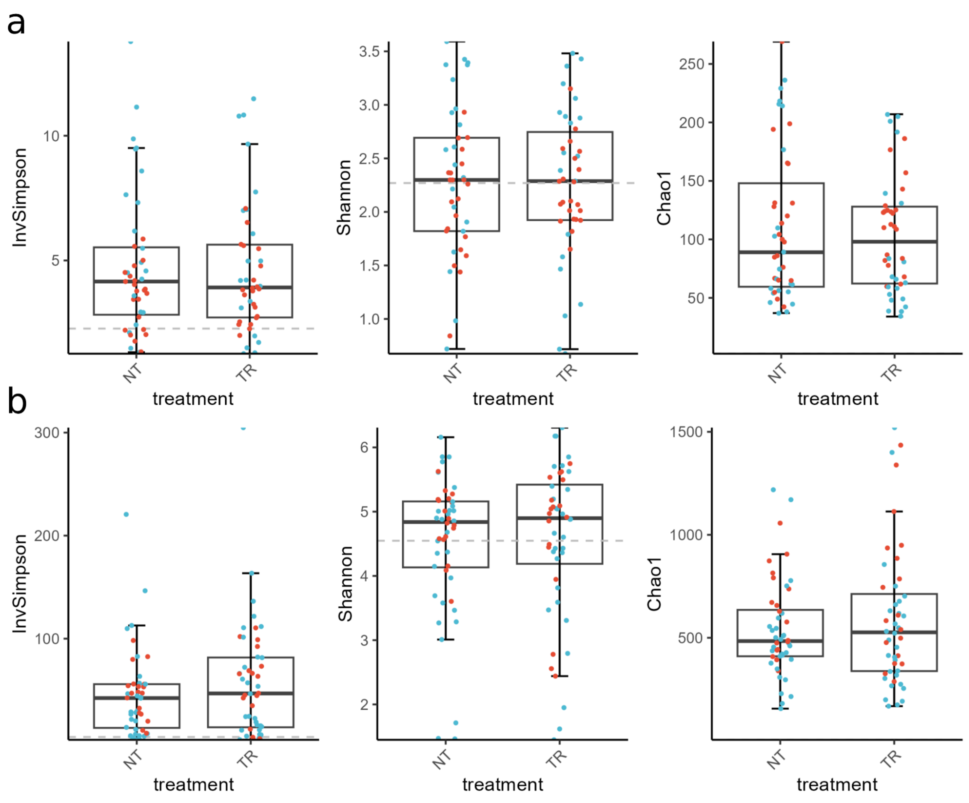

Figure S1: Bacterial (a) and fungal (b) diversity indices across treated (TR) and non-treated (NT) samples. Blue points indicate the samples from indoor individuals, the red dots the ones from outdoor individuals.

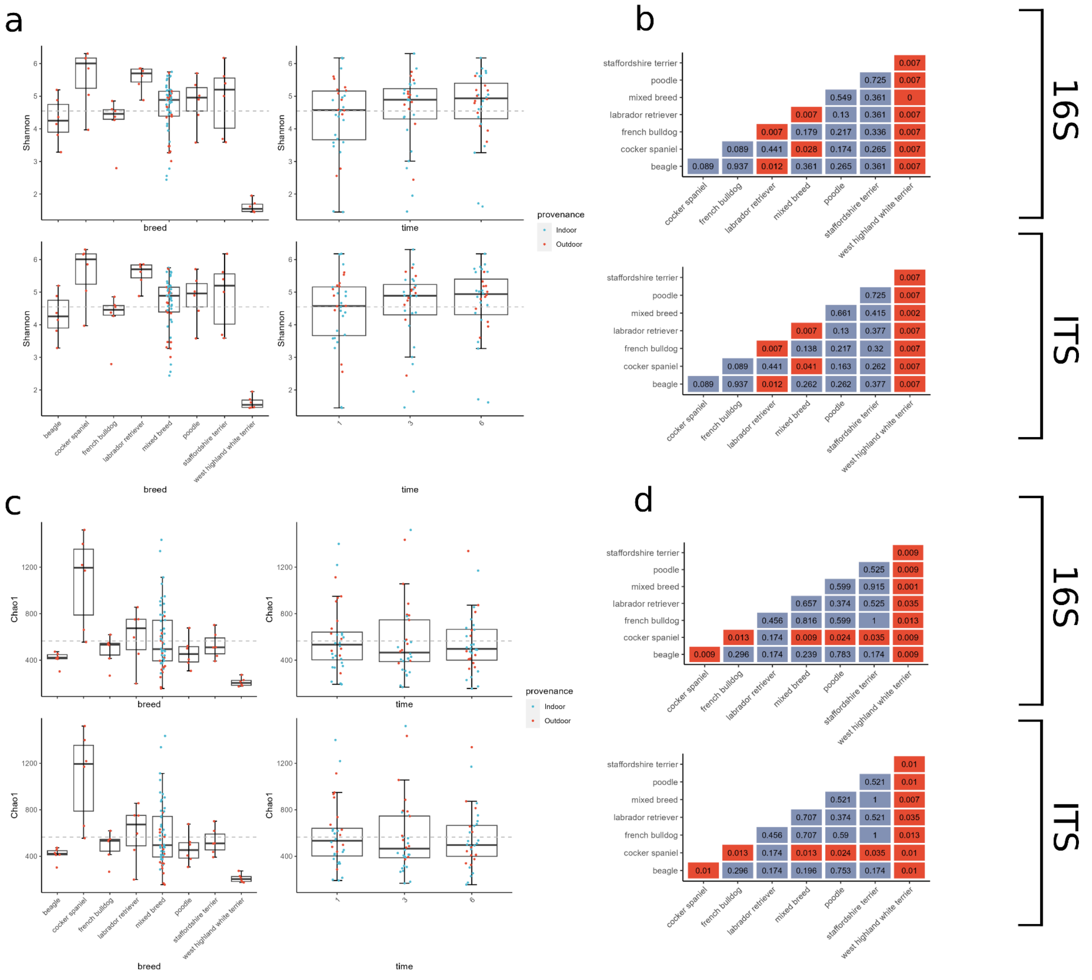

Figure S2: Shannon (a) and Chao1 (b) diversity indices across bacterial and fungal samples. On the right the results of Wilcoxon tests, in red the significant results. Colors of dots in the boxplot indicate the dogs' provenance.

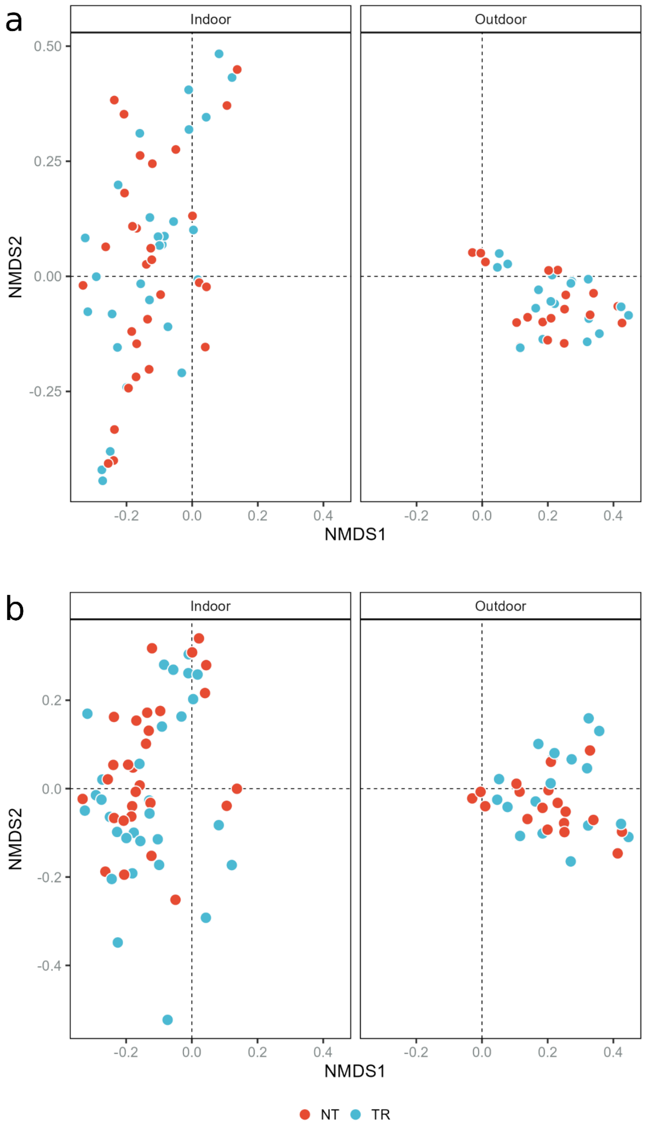

Figure S3: Bacterial (a) and fungal (b) NMDS plots split by provenance. Colors show samples from treated (blue dots) and non-treated (red dots) sides.
